# Families’ opinions about their involvement in care during hospitalization: a mixed-methods study

**DOI:** 10.1186/s12912-024-02664-8

**Published:** 2025-01-08

**Authors:** Josien M. Woldring, Wolter Paans, Reinold O. B. Gans, Hinke M. van der Werf, Marie Louise Luttik

**Affiliations:** 1 Research Group Nursing Diagnostics, Family Care & Family Nursing, School of Nursing, Hanze University of Applied Sciences, Petrus Driessenstraat 3, Groningen, 9714 CA The Netherlands; 2 Department of Internal Medicine, University Medical Center Groningen, University of Groningen, Hanzeplein 1, Groningen, 9713 GZ The Netherlands; 3 Department of Critical Care, University Medical Centre Groningen, PO Box 30.001, Groningen, 9700 RB The Netherlands

**Keywords:** Patient and family-centered care, Family involvement, Family Nursing, Hospital care, Hospitalization, Nursing care, Healthcare professionals, Collaboration, Role alignment

## Abstract

**Background:**

Healthcare professionals in the hospital setting frequently assume primary caregiving responsibilities, which often leads family members to perceive this as standard practice during hospitalization. This dynamic may create a gap between actual and desired levels of family involvement. The aim of this study is to explore the opinions of families about their involvement in care during the hospitalization of a relative.

**Methods:**

A sequential explanatory mixed-method study design was used, where quantitative data collection is followed by qualitative data collection for a deeper understanding of the quantitative findings. Data were collected between November 2023 and April 2024 across 15 wards in a university hospital in the north of the Netherlands. 153 family members of hospitalized patients completed the adapted Families’ Importance in Nursing Care–Families’ Opinions questionnaire, providing valuable quantitative data. Twenty-three of these family members were subsequently interviewed to gather qualitative insights. Data were analyzed sequentially, with the quantitative results guiding qualitative data collection. The two types of data were integrated to draw comprehensive conclusions about the significance of family involvement during hospitalization. The study adhered to the guidelines of the Good Reporting of A Mixed Method Study (GRAMMS).

**Results:**

The questionnaire scores indicate a high willingness for involvement in care during hospitalization. Subsequent in-depth interviews led to the development of a model demonstrating that this involvement is sequentially related to the themes of acknowledgement, alignment, and collaboration.

**Conclusions:**

Family members expressed a need to be acknowledged by healthcare professionals as partners in care. Role agreements and information sharing during hospital care should be aligned to achieve effective collaboration between family members and healthcare professionals.

**Supplementary Information:**

The online version contains supplementary material available at 10.1186/s12912-024-02664-8.

## Background

Societal shifts toward an aging population and the increased prevalence of chronic diseases, combined with a decline in the number of healthcare professionals, have led to a greater reliance on family members for caregiving at home [1]. Beyond providing direct care, family members are involved in the care process of their family member (the patient) at home and feel responsible for them, even during hospitalization. Family members nowadays take on complex tasks, from managing medications to coordinating care, while also maintaining the patient’s emotional well-being [2]. Their knowledge of the patient’s needs and preferences becomes crucial during transitions to hospital care [3]. In hospitals, family members can serve as advocates and partners, offering insights into the patient’s condition and supporting healthcare teams. Their involvement improves communication, enhances care continuity, and contributes to better outcomes and satisfaction [4].

In this study, we adopted the broad definition of family members from Benzein et al. [5], describing them as a self-defined group of individuals considered significant for the patient, regardless of blood ties or law.

Hospitalization can have physical, emotional, cognitive, and social effects on both the patient and family members [6]. Illness and hospitalization significantly impact family members, who can become overwhelmed by the demands of caring for the ill patient [7]. A such, healthcare professionals should be aware of the family members’ situation and desire to be involved in the patients’ care during hospitalization.

The length of hospital stays is shortening, resulting in patients frequently being discharged despite not yet having fully recovered [8]. Several studies suggest that the preparedness of family members for their caregiving role highly depends on the involvement and support they did receive from healthcare professionals during hospitalization [9, 10]. Better continuity of care and preparedness for the home situation can be ensured when family members are actively involved and supported by healthcare professionals, including during the hospitalization process [4].

The institute for Patient- and Family-Centered Care advocates for emphasizing open communication, shared decision-making, and respectful partnerships between patients, family members, and healthcare professionals [11]. Achieving open communication and partnership requires an understanding of both family members’ and healthcare professionals’ opinions regarding the role of family members in patient care. Establishing clear role expectations is crucial; it fosters a more harmonious and effective care environment during hospitalization and better preparedness for the home situation [12].

Family members often lack a clearly defined role during hospitalization, making it challenging to delineate the boundaries between the responsibilities of healthcare professionals and those of family members. This ambiguity can lead to healthcare professionals taking over care in the hospital setting without aligning with the existing care relationship between family member and patient [13, 14].

Benzein et al. [5] developed an instrument to investigate nurses’ attitudes toward family involvement in care. This instrument explores whether nurses are willing to acknowledge family members for their valuable knowledge about the patient and their willingness to integrate family members as not only caregivers but also part of the care team [5]. Using this instrument, Hagedoorn et al. [15] found that nurses who maintain a positive attitude toward involving family members as partners in care are more inclined to communicate and collaborate more effectively with family members.

Benzein et al.’s instrument [5] has been adapted by Woldring et al. [16] to measure the family perspective on their involvement in care. The findings of that study highlight that family members of patients receiving homecare desire active involvement in nursing care and seek acknowledgment in care planning [16].

Nevertheless, healthcare professionals in the hospital setting frequently assume primary caregiving responsibilities, which often leads family members to perceive this as standard practice during hospitalization. This dynamic may create a gap between actual and desired levels of family involvement. As such, broad and deep insight into the opinions of family members about their involvement in care during hospitalization of their family member is needed.

Therefore, this study aimed to provide insight into family members’ opinions about their involvement in the care of a hospitalized relative. Family involvement is relevant for hospitalized patients admitted to all medical specialties. In this study, we focused on family members of patients admitted to either surgical or medical departments. When we refer to healthcare professionals, we mean both nurses and physicians; these are the professionals consistently involved during the hospital stay. Although the questionnaire used in this study originally focused on nursing care and was thus named *family importance in nursing care*, for the purpose of this research, we extend the concept of nursing care to comprise the overall hospital care provided by nurses and physicians.

## Methods

### Design

This study employed a sequential explanatory mixed-method design [17], as summarized in Table 1. A two-phase approach was utilized, where the findings from the quantitative data collection in Phase 1 directly informed the qualitative data collection in Phase 2, enabling a more nuanced exploration of the results. Phase 1 involved the use of a questionnaire to provide a broad overview of family members’ opinions regarding their involvement in hospital care. Insights from Phase 1 were used to develop the interview guide for Phase 2, allowing the qualitative interviews to delve deeper into and refine the themes and patterns identified in the quantitative data*.* This study adhered to the guidelines of the Good Reporting of A Mixed Method Study (See repository) [18].

**Table 1:** Mixed-method sequential explanatory design procedure

Phase	Procedure	Product
**1. Quantitative data collection**	-Cross-sectional survey (*n* = 153) Distributed in: Hospital: 1 Wards: 15 -Questionnaires distributed *n* = 300 -Response of Family members *n* = 153	Numeric data
**2. Quantitative data analysis**	-SPSS for Windows (release 28.0.1.1) -Descriptive statistics -Psychometric statistics: Internal consistency and reliability	Results from analyses Score per questionnaire item Cronbach’s alpha
**3.Connecting quantitative and qualitative phases**	-Purposefully selecting participants based on maximal variation (age, gender, level of education, type of relationship with the patient, number of hours of caregiving, working status, admission department of the patient and scores on the FINC-FO) -Develop an interview guide based on quantitative analysis. Analysis provides a global overview of the questionnaire results, rather than feedback on individual scores	Interview guide
**4. Qualitative data collection**	-Select and approach family members by mail or telephone -Individual in-depth face to face or online interviews (Microsoft Team) -Make field notes -Transcribe interviews into text	23 interviews Audio recorded Transcripts
**5. Qualitative data analysis + start data integration**	-ATLAS ti.24 software -Coding and thematic analysis using subscales FINC-FO -Design a model of the themes	Codes and themes Visual model
**6. Integration of the qualitative and quantitative results**	- Interpretation and explanation of the quantitative and qualitative results	Discussion Implications Future research

### Study setting and sampling (inclusion and exclusion criteria)

Family members of patients hospitalized at the University Medical Centre Groningen were approached to complete a questionnaire about their opinion of involvement in care during hospitalization. Family members were eligible to participate if they were (1) over 16 years of age and (2) proficient in the Dutch language. Family members of patients in the critical care, pediatric, or psychiatric wards were excluded for this research. Previous studies show that families of patients in these wards often have a different role because of the patient’s clinical situation [9, 19–21].

Family members of patients admitted to 15 wards (6 surgical and 9 medical) of the University Medical Centre Groningen were asked to complete a questionnaire. To ensure a representative sample of the number of wards and variety of variables in the questionnaire, the goal was to collect 300 completed questionnaires (150 surgical and 150 medical). To achieve this, approximately 20 questionnaires were distributed per ward. Ultimately, 155 family members completed the questionnaire, a response rate of 52%.

At the end of the questionnaire, family members were asked if they would be willing to participate in an interview that would expand on topics addressed in the questionnaire. Of the 155 participants who completed the questionnaire, 47 agreed to be contacted for an interview. We purposefully selected individuals who varied in term of age, gender, relationship to the patient, paid employment, caregiving tasks, and hospital department. Enrollment in Phase 2, the qualitative part of our study, ceased after 23 interviews, when thematic saturation was reached. This was determined through an iterative process of conducting and analyzing interviews, where no new themes or insights emerged, ensuring the data comprehensively captured family members’ perspectives on care involvement.

### Quantitative data collection and analyses (Phase 1)

#### Instrument

We used the Family Importance in Nursing Care–Families’ Opinions (FINC-FO) questionnaire [16]. This questionnaire has been validated through deployment among family members within the home care setting [16]. In the current study, we removed two specific statements, as well as the entire *family as a burden* subscale, comprising four statements, due to their low psychometric performance [16]. Additionally, recognizing that family members in the hospital interact with not only nurses but also physicians regarding diagnosis and treatment plans, we introduced a new statement in the questionnaire centered on family involvement in decision-making regarding diagnosis and treatment plans. The adapted FINC-FO questionnaire consequently comprises 20 statements distributed across three subscales, each assessed using a 5-point Likert scale (1 = strongly disagree–5 = strongly agree). These subscales explore *family as a resource in care* (9 items), *family as a conversational partner* (7 items), and *family as its own resource* (4 items) [18] (See supplemental file 1).

#### Data collection

In November and December 2023, one family member of each patient hospitalized in the University Medical Centre of Groningen was approached to complete the FINC-FO questionnaire. Questionnaires were distributed exclusively in paper format to ensure ease of use. Although the distribution was initiated by nurses working on the respective wards, the high workload and competing priorities often limited their availability to actively hand out the questionnaires. As a consequence, nursing students played a significant role in the distribution process, primarily during visiting hours. Despite these efforts, not all patients had family members who could be reached due to e.g. absence of family visitors during the times nursing students were present on the ward. Also some patients did not have family members visiting them at all.

Demographic characteristics such as age, gender, level of education, type of relationship with the patient, number of hours of caregiving, working status, and admission department of the patient were subsequently collected. The entire data collection process took 2 months.

#### Data analyses

Data were analyzed using SPSS for Windows (release 28.0.1.1). Descriptive statistics were used to describe the study population and the responses to the FINC-FO questionnaire on item level. Higher scores indicate more positive opinions about family involvement. Education level was categorized as high (tertiary education), middle (secondary education), or low (primary education), and relationship to the patient was merged into three categories: spouse, child (including son-in-law and daughter-in-law), and other.

Cronbach’s alpha was used as an indicator of the internal consistency and reliability for both the overall FINC-FO questionnaire and its individual subscales. An alpha value of 0.70 or higher is generally considered acceptable, while values of 0.80 or higher are considered excellent [22].

### Qualitative data collection and analyses (Phase 2)

#### Data collection

Qualitative data were collected through semi-structured interviews to explore family members’ perspectives on their involvement in care during hospitalization. Participants were purposively selected from those who completed the quantitative phase and had indicated a willingness to participate in an interview, ensuring diversity in demographics and levels of involvement (see Table 1). An interview guide [18] (see supplemental file 2), informed by quantitative findings, was refined through pilot testing to ensure clarity and relevance. Pilot testing also helped prepare the interviewer by allowing them to practice the interview process, identify and adjust any issues with question wording before the full study. All interviews were conducted by the interviewer, JW, who had no prior relationship with any of the family members interviewed. JW had prior experience with qualitative research in a previous study, and further developed interview expertise by attending a dedicated interview training course.

A total of 23 interviews were conducted over two months, lasting approximately 45 min each, either online via Microsoft Teams, face-to-face at home, or in the hospital, depending on participant preferences. Interviews were audio-recorded, supplemented by field notes, and preceded by an explanation of the study's purpose and assurance of interviewer neutrality. This comprehensive process aimed to provide rich, in-depth insights into the underlying motives and rationale behind family members’ opinions.

#### Data analyses

The recordings of the interviews were transcribed verbatim. The interviews were analyzed following the steps of thematic analysis: becoming familiar with the data, generating initial codes, searching for themes, reviewing themes, defining and naming themes, and reporting [23]. Researchers JW and HW, who have experience in analyzing interviews from previous studies, initially worked separately using an inductive approach, systematically noting all topics related to family involvement during hospitalization as mentioned by the participants. These topics were then discussed by the research team, all of whom have experience in conducting qualitative research. A deductive analysis was employed to categorize the topics according to the subscales of the FINC-FO questionnaire *(family as a resource in care, family as a conversational partner,* and *family as its own resource).* Some topics, however, did not fit under the subscales. As a result, the entire research team collaboratively grouped the topics into overarching themes [18].

#### Rigor

Lincoln and Guba (1985) identify credibility, transferability, dependability, and confirmability as foundational criteria for establishing trustworthiness in qualitative research [24]. Each criterion addresses a specific aspect of research rigor and ensures that findings are robust and meaningful. Credibility refers to the accuracy and truthfulness of the researchers' representation of participants' views and experiences [25]. To enhance credibility in this study, methodological triangulation was employed. Specifically, two researchers (JW and HW) independently analyzed the interview transcripts, thereby reducing potential individual biases. Additionally, iterative reflection rounds were conducted among the research team to discuss and refine interpretations. These discussions continued until consensus was reached on the coding framework and final analysis, strengthening the reliability of the findings. Transferability pertains to the extent to which the findings of a study can be applied to other settings.

Dependability focuses on the stability and consistency of the research process over time [25]. To ensure dependability, the study design, data collection, and analysis procedures were meticulously documented. This documentation included a comprehensive audit trail that outlined all steps of the research process, ensuring it was logical, traceable, and transparent. Peer debriefings and regular team discussions further contributed to maintaining consistency and addressing potential methodological discrepancies.

Confirmability ensures that the interpretations and conclusions drawn by researchers are firmly grounded in the data, minimizing the influence of personal biases or preconceptions [25]. To enhance confirmability in this study, verbatim quotes from participants were incorporated into the Findings section, providing direct evidence to support the themes and interpretations. Furthermore, a self-reflective approach was adopted. Researchers maintained detailed field notes after each interview, recording personal biases, emotional responses, and critical insights. These reflexive notes served to heighten awareness of subjective influences and allowed for continuous adjustment of the analysis process to remain aligned with the data [26].

### Integration

The initial integration was achieved by linking the methods of data collection and analysis through connecting and building [27]. Quantitative findings were used to design an interview guide for the qualitative phase and to purposefully sample family members who varied in terms of age, gender, relationship to the patient, paid employment, caregiving tasks, and hospital department for interviews. Integration at the interpretation and reporting level was subsequently facilitated through a narrative using a contiguous approach [27]. Although quantitative and qualitative findings were reported in separate sections, they were integrated during the interpretation of the qualitative topics and themes. This integration of findings contributed to a deeper understanding of the quantitative results.

### Ethical considerations

This study was approved by the Medical Ethics Committee of the University Medical Centre of Groningen (PaNaMaID 15878). Eligible family members received information about the study and provided written informed consent. All legal aspects regarding privacy and confidentiality were ensured.

## Results

### Quantitative results

Of the 155 completed questionnaires, two were excluded: one questionnaire was completed by a family member younger than 16 years of age, and one had incomplete data. As a result, 153 questionnaires remained for data analysis.

#### Participants’ characteristics

The age of the participants (*n* = 153) ranged from 18–93 years, with a mean age of 52 years. Most participants were female (63%), and 50% had a middle-level education. Nearly all (97%) reported the Netherlands as their country of origin. Four participants originated from outside Europe: two from Indonesia, one from Iraq, and one from Curacao. More than half of the participants were spouses of a hospitalized patient. A significant proportion of the participants had paid employment or structural obligations (71%), and one-third (31%) provided care at home (Table 2). Approximately equal distribution was observed across hospital departments: 46% of participants were family members of the surgical department, while 54% were family members of patients in the medical department.

**Table 2:** Characteristics of the quantitative survey research participants (*n = 153)*

	**Mean (SD)**
**Age (years)**	52 (5.3)
	**N (%)**
**Gender**	
Female	96 (62.7)
Male	56 (36.6)
**Education level**	
Low	28 (18.4)
Middle	70 (46.1)
High	54 (35.3)
**Country of origin**	
Netherlands	148 (96.4)
Out of Europe	4 (2.6)
**Relation to the patient**	
Spouse	82 (53.6)
Child (in-law)	46 (30.1)
Other	25 (16.3)
**Paid employment/Structural obligations**	
Yes	109 (71.2)
No	44 (28.8)
**Caregiving at home**	
Yes	48 (31.4)
No	105 (68.6)

#### FINC-FO questionnaire scores

Table 3 shows the total score on the FINC-FO questionnaire and its subscales. The total score of 76.5 (SD 10.6; range 20–100), as well as the scores on the subscales *family as a resource in care* and *family as a conversational partner*, were greater than 75% of the maximum possible score.

**Table 3:** Scores on the subscales of the Families’ Importance in Nursing Care–Families’ Opinions questionnaire (*n* = 153)

Subscales	MEAN (SD)	MIN–MAX	THEORETICAL RANGE
Family as a resource in care: 9 items	34.5 (5.3)	18**–**45	9**–**45
Family as a conversational partner: 7 items	27.7 (4.2)	16–35	7–35
Family as its own resource: 4 items	14.2 (3.0)	4**–**20	4**–**20
**Total**	**76.5 (10.6)**	**40–100**	**20–100**

Table 4 illustrates the response percentages per subscale at the item level and the Cronbach’s alpha as an indicator of internal consistency and reliability. For both the individual subscales and the overall FINC-FO questionnaire, Cronbach’s alpha is 0.80 or higher, indicating that the FINC-FO has excellent internal consistency. Additionally, no item-item correlations exceeded 0.80, indicating that there were no duplicative items within the subscales.

**Table 4:** Results of the Family Importance in Nursing Care – Families Opinions (*n* = 153)

	**Percentages per category of response**^**a**^** (%)**					**α if item** **deleted**
Subscales	**1**	**2**	**3**	**4**	**5**	
Family as a resource in care						
1. My presence when my family member receives care is meaningful	1	2	14	41	41	.78
2. My presence as a family member eases the workload of nurses and physicians	5	7	45	31	12	.76
3. It is important to me that I am invited to take an active part in the planning of care	5	6	25	41	24	.76
4. It is important to me that I am present when care is provided	2	11	31	39	18	.77
5. It is important to me to discuss how I can take an active part in care	1	7	20	47	25	.76
6. It is important to me to be involved in the decision-making process regarding diagnosis and treatment	1	3	14	33	50	.79
7. It gives me a feeling of being useful when I am involved in care	1	5	26	43	25	.77
8. I possess a lot of worthwhile knowledge about my family member that nurses and physicians can use in their work	7	7	25	35	26	.78
9. It is important to me that nurses and physicians spend time with me	1	3	24	46	26	.80
Cronbach’s alpha total subscale						.80
Family as a conversational partner						
1. It is important to me that nurses and physicians know who the patient’s family members are	1	3	20	47	28	.81
2. It is important to me that I am invited to take an active part in caring for my family member	2	11	28	36	23	.77
3. It is important to me that I am invited to a conversation at the start of care	1	5	20	39	35	.76
4. A conversation with me as a family member at the start of care will save nurses and physicians time in their work in the future	1	12	35	31	20	.76
5. It is important to me that I am invited to a conversation at the end of care	1	7	22	36	34	.76
6. It is important to me that I am invited to a conversation when my family member’s situation changes or takes a turn for the worse	0	1	3	29	67	.79
7. It is important to me that I am regularly invited to a conversation on the progress (planning) of care	1	4	28	40	26	.75
Cronbach’s alpha total subscale						.80
Family as its own resource						
1. It is important to me that nurses and physicians ask me how they can support me	2	9	41	35	14	.76
2. It is important to me that nurses and physicians encourage me to cope with the situation myself as best as I can	5	6	41	33	14	.71
3. It is important to me that nurses and physicians see me as a cooperating partner	5	8	33	37	16	.80
4. It is important to me that nurses and physicians help me cope with the situation as best as I can	1	6	32	40	19	.70
Cronbach’s alpha total subscale						.80
Cronbach’s alpha for the total FINC-FO						.89

^a^1 = Strongly disagree, 2 = Disagree, 3 = Neutral, 4 = Agree, 5 = Strongly agree

The scores of 4 (agree) and 5 (strongly agree) for items of the 3 subscales are discussed below, providing further insights into family members' perspectives on their involvement in care.

##### Family as a resource in care

A significant proportion of the family members indicated that their presence during care is meaningful (82%) and that it was important for them that healthcare professionals spend time with them (72%). Family members wanted to be involved in the decision-making process regarding diagnosis and treatment plans (85%) and wanted to discuss how to take an active part in care (72%). More than half of the family members indicated that they possess a great deal of worthwhile knowledge about their family member that healthcare professionals can use in their work (61%). Family members considered it important to be invited to take an active part in the planning of care (64%) and be present when care is provided (57%). Most family members were neutral about the statement regarding the “presence of family members eases the workload of healthcare professionals.”

##### Family as a conversational partner

Seventy-five percent of the family members found it important that healthcare professionals identify those persons who belong to the family. While 74% valued being invited to a conversation at the start of care, only 51% felt that this would be time efficient. Additionally, family members expressed a desire to be invited into conversation when changes in the clinical situation occur (96%), for regular progress updates (66%), and at the end of the hospital stay (70%).

##### Family as its own resource

Nearly half of the respondents (49%) found it important to be asked how they as a family member could be supported, and 59% wanted support from healthcare professionals in coping with the situation. About half of the family members (53%) valued being recognized by healthcare professionals as a collaborating partner. More than one-third of the family members remained neutral on the four items of this subscale.

### Qualitative results

#### Participants’ characteristics

Twenty-three family members who completed the FINC-FO questionnaire were subsequently interviewed. The characteristics of each interviewed family member and their scores on the FINC-FO are presented in Table 5.

**Table 5:** Characteristics of the interview respondents and their scores on the Families’ Importance in Nursing Care – Families’ opinions (*n* = 23)

Respondent number	Age Range (years)	Gender	Education Level	Relation	Paid employment	Caregiving at home	Hospital Department	Score FINC-FO Total^a^ *Theoretical* *Range* *20–100*	Score Subscale RC^b^ *Theoretical Range* *9–45*	Score Subscale CP^c^ *Theoretical Range* *7–35*	Score Subscale OR^d^ *TheoreticalRange* *4–20*
1	35–40	Female	Low	Child	Yes	Yes	Surgical	83	41	29	13
2	55–60	Male	High	Child	No	No	Medical	86	42	31	13
3	45–50	Male	Middle	Spouse	Yes	No	Medical	86	39	31	16
4	40–45	Male	Middle	Spouse	Yes	No	Medical	89	41	32	16
5	50–55	Male	Low	Spouse	Yes	Yes	Medical	69	29	25	15
6	65–70	Male	Middle	Spouse	No	Yes	Medical	92	41	35	16
7	45–50	Female	High	Child	Yes	No	Medical	84	35	34	15
8	60–65	Male	Middle	Child	Yes	No	Surgical	88	40	31	17
9	70–75	Female	High	Spouse	No	No	Medical	82	40	29	13
10	30–35	Female	High	Child	Yes	No	Surgical	70	35	26	9
11	80–85	Male	Low	Spouse	No	Yes	Medical	81	34	31	16
12	50–55	Female	Middle	Spouse	Yes	No	Surgical	82	35	31	16
13	45–50	Female	High	Spouse	Yes	Yes	Medical	80	36	29	15
14	70–75	Female	High	Spouse	No	Yes	Surgical	91	41	34	16
15	65–70	Male	High	Spouse	No	No	Medical	75	34	29	12
16	40–45	Male	High	Child	Yes	No	Surgical	91	41	33	17
17	35–40	Male	High	Child	Yes	No	Surgical	84	38	33	13
18	30–35	Female	Middle	Child	Yes	No	Medical	72	27	25	20
19	40–45	Female	High	Spouse	Yes	No	Medical	80	36	29	15
20	55–50	Female	High	Spouse	Yes	No	Surgical	84	37	32	15
21	50–55	Female	Middle	Child	Yes	No	Surgical	76	31	29	16
22	25–30	Female	High	Child	Yes	No	Medical	80	37	28	15
23	65–70	Female	High	Spouse	No	No	Surgical	65	31	28	6

^a^Families’ Importance in Nursing Care – Families’ Opinions – 20 questions

^b^Subscale family as a resource in care – 9 questions

^c^Subscale family as a conversational partner – 7 questions

^d^Subscale family as its own resource – 4 questions

Of the 23 family members, the mean age was 52 years. There were approximately equal distributions of gender (13 female versus 10 male), relationship to the patient (13 spouse versus 10 child), and admission department (10 surgical versus 13 medical). Paid employment or structural obligations (16 yes versus 7 no) and caregiving at home (6 yes versus 17 no) showed distributions like those in the questionnaire sample.

#### Interview findings

**Fig. 1:**
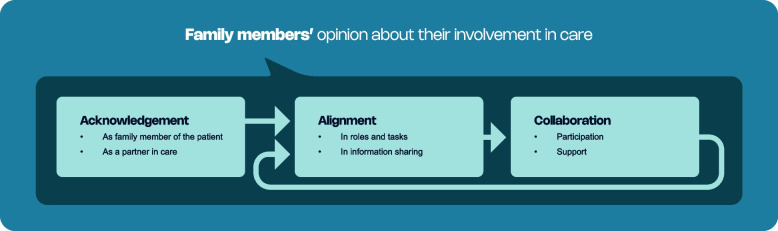
Sequential themes of family involvement as perceived by family members of hospitalized patients

Three themes were identified from the interviews to explain the opinions of family members about their involvement during hospitalization more in depth: (i) acknowledgement, (ii) alignment, and (iii) collaboration. As seen in Fig. 1, the themes follow a sequential order. Notably, many of the interviewed family members regarded family involvement as a critical issue and were pleased that it received attention. Moreover, several conversations revealed significant emotional responses as family members recounted their experiences during the hospital stay. Additionally, many family members, particularly those of older patients, noted that their situations were unique due to the patient having multimorbidity, necessitating interactions with various specialists.

### Theme 1. Acknowledgement

Family members indicated that they are an important asset in patient care and want to play a role during hospitalization. Acknowledgment by healthcare professionals is seen as an essential first step.

#### Being a family member of the patient

Family members feel responsible for the patient and want to continue supporting them and thus wish to be seen and listened to by healthcare professionals.

*That healthcare professionals should not just focus on the tasks at the bedside, but also be aware of who else is present. Noticing if there’s a new face and understanding their relationship to the patient—that is important.* (Respondent 17).

Family members believe that attention should focus on not only the patient but also their significant others. They expressed a desire to get to know the healthcare professionals and build a trusting relationship with them.

#### Being a partner in care

Family members also expressed a desire to be acknowledged as partners in care. Emphasizing the importance of a positive hospitalization experience for the patient, they indicated their willingness to contribute to that goal.

*You just want the hospitalization to go as smoothly as possible for all parties, and any contribution as a family member to ensure that you are willing to make. That is how I see involvement or how I would like it to be.* (Respondent 13).

Family members also indicated that they bring valuable experience and knowledge about the patient, insight that healthcare professionals may benefit from.

*You sometimes feel somewhat ignored, even though as a caregiver you can provide them (healthcare professionals) with a lot of important information.* (Respondent 20*).*

Family members noted that they continue to feel responsible for the patient and cannot easily relinquish this responsibility during hospitalization.

*Involvement and care do not stop at the door of a hospital room.* (Respondent 14).

*The nurses and everyone at the hospital have so much knowledge and experience that you may let go of everything. But you never do.* (Respondent 5).

### Theme 2. Alignment

After acknowledgment by healthcare professionals, the next key step for family members is the alignment of roles, tasks, and information processes. Family members indicated that it is essential to clearly define and agree on the roles they can and wish to take on during hospitalization. An important prerequisite mentioned in every interview was that there must also be alignment in information sharing, so that family members receive all necessary information about the illness, care, and organizational processes and can also share relevant information with healthcare professionals.

#### Role alignment of family members

The way family members want to be partners in care during hospitalization often relates to the autonomy of both the patient and the family members. Spouses indicated that as a couple they prefer to manage everything together and maintain their autonomy before involving their children. Spouses often expressed that as a couple they do not want to burden their children unless it is absolutely necessary. Children, however, stated that they are often willing to support their parents (the patient) during hospitalization. However, as noted by Respondent 7, when the patient still has a partner, children sometimes find it challenging to be involved in the care of their parents.

*I also had to get used to that role because my parents have always been very self-reliant and independent. And then suddenly you apparently have a role. You don’t want to impose yourself on them.* (Respondent 7).

Role fulfillment as a partner in care should be aligned with the wishes and capabilities of the patient, family, and healthcare professionals. Family members consistently noted that as patients become frailer and more ill, their role as a partner in care becomes larger and more crucial, particularly regarding communication.

*He [father] is so preoccupied with his illness that he no longer has the ability to understand what a stranger [healthcare professional] is asking from him.* (Respondent 8).

Additionally, family members indicated that taking over care during hospitalization may lead to a shift in roles within the family, which some family members find undesirable.

*Then I no longer feel like a spouse, but more like a caregiver and helper.* (Respondent 12).

The physical ability of the family member, the home situation (i.e., other roles must be fulfilled), the family situation (division of care within the family), work (flexibility), the duration of the hospital stay, and the distance to the hospital were all mentioned as factors influencing the role of family members as partners in care during hospitalization.

#### Information-sharing alignment

An important condition for family members to perform a role as partners in care, mentioned by all family members, is staying informed about the patient’s situation. As Respondent 10 highlighted, family members want to be informed about the current situation and (future) developments during hospitalization, including clear expectations for discharge and preparation for the home situation afterward.

*I think it also involves the expectation process. What is expected of you and how you can contribute to the care process. Yes, especially when the patient goes home again. What can we do now to ensure it goes well at home later?* (Respondent 10).

Families indicated a preference to be present in discussions between the patient and healthcare professionals, either in person or remotely, to receive and provide information. Reasons included the patient’s inability to recall or accurately convey all information and the unavailability of the appropriate healthcare professional to provide or repeat information later.

*When I visit my father, which is often in the evening for me because I work during the day, they sometimes say, “Yes, I don’t know the answer to that question, you should call during the day tomorrow because then the person who can provide the answer will be present.”* (Respondent 1).

Family members indicated that receiving timely and proactive information is beneficial for staying well informed and being able to participate in decision-making. A majority of the family members perceived the need to have to ask repeatedly for information as a barrier. As noted by Respondent 2, family members acknowledged that healthcare professionals are busy and thus found it challenging to burden them with additional questions.

*We are also may be too modest about this [asking questions]. We could have walked up to the reception desk more often, saying, “We do not quite understand something, can you explain it to us?” But we are also left with conflicting thoughts like, “They are busy, oh well, we will figure it out.” (*Respondent 2).

Clarity in the care process during hospitalization is also regarded as important. Understanding how a department operates and what is feasible for family members enables them to align their roles and tasks during hospitalization.

*If you are there when the doctor comes by, you may get a thorough explanation and the opportunity to ask questions. But if you are not there, it becomes unclear how you can still get the necessary information.* (Respondent 17).

It is also crucial for family members to anticipate their role in a timely manner and manage everything related to it. Family members indicated that they cannot always come ad hoc but need time to organize things at home and at work.

Furthermore, as highlighted by Respondent 21, family members emphasized the importance for healthcare professionals to have a clear understanding of the patient’s (home) situation, enabling a broader perspective that encompasses not only the medical condition but also the patient’s social situation, as well as their perspective on their goals in life.

*When my father was lying there, I thought, “Oh, he looks just like an old man” … It’s important for them [healthcare professionals] to know how someone is at home and has still been active in their life.* (Respondent 21).

Family members perceive it as their role to share this knowledge and their experiences with the healthcare team and have them integrate this information into the decision-making process.

### Theme 3. Collaboration

Families indicated that when they are acknowledged as partners in care and their roles and the information process are well aligned, they become invaluable collaborators in participating and supporting in the patient’s care during hospitalization. In addition, family members recognized that in this collaboration, attention must be paid to their own wellbeing as family members to sustain their role as partners in care effectively.

#### Participation of family members

Family members often mentioned acting as a mediator, bridging the gap between patient and healthcare professional, ensuring that communication occurs at the same level, and everyone is on the same page.

*My role is essentially to facilitate this connection repeatedly during the conversation [making a connecting gesture]. Because somehow, yes, I do not know how to say this, but they communicate in a different way, at a different level.* (Respondent 13*).*

Children of hospitalized parents reported translating, clarifying, explaining, or contextualizing information for the patient, leading to a better understanding by the patient and facilitating shared decision-making.

Many partners indicated that they navigate the illness and hospitalization process together with the patient, and therefore, they want to hear conversations jointly so they can review and discuss potential treatment decisions as a pair.

*We (patient and spouse) will weigh the pros and cons together and ensure that we both agree on the decision that is made.* (Respondent 9).

Most family members indicated that they are willing to take on caregiving tasks for the patient during hospitalization, provided this is coordinated with the wishes, possibilities, and capacities of all parties involved. Family members mentioned that they do not yet see this role being implemented in practice and are also hesitant to take the initiative themselves.

*I saw how busy the nurses were, so I said, “I am here, so I can help out if you need me.” But they told me it wasn’t necessary [Family member was staying in a hotel and had to wait until visiting hours].* (Respondent 20).

As noted by Respondent 3, family members acknowledged that taking over caregiving tasks may have benefits for both patient and healthcare professional.

*If your family member is really struggling, because he or she is very sick, how nice is it then to have a loved one help you? Rather than having a stranger [healthcare professional] come and, for example, wipe your bottom.* (Respondent 3).

Additionally, family members noted that when they receive guidance and trust from health care professionals in performing caregiving tasks during hospitalization, it increases their confidence in the discharge process.

#### Receiving support of healthcare professionals

Most family members indicated that the patient is central to the care process. However, they also value it highly when healthcare professionals pay attention to the family. Although family members may share their concerns within their network for support, hearing the question “How are you?” from a healthcare professional was perceived as valuable.

*They asked us when we were waiting in the hospital for so long, “How are you all doing?” And then we all burst into tears. We just couldn’t hold it in anymore. That was the only time throughout this hospitalization that someone asked us how we were doing.* (Respondent 16).

Family members reported that the illness or hospitalization of a significant other had a significant impact on them, both mentally and practically. Understanding and guidance from healthcare professionals was reported as enabling family members to effectively maintain their chosen roles, ensuring continuous (role) alignment and collaboration with the healthcare professionals in patient care during hospitalization.

### Integration through interpretation and reporting

The qualitative findings from this study provide a valuable extension of the subscales of the FINC-FO questionnaire, offering deeper insights into the perspectives and needs of family members during hospitalization. The quantitative findings from Phase 1 significantly influenced the design of the interview guide for Phase 2, facilitating a more targeted exploration of key themes.

The *family as a conversational partner* subscale highlighted the importance of family members being acknowledged and engaged in discussions with healthcare professionals. Interviews further explored why such recognition is significant, when it becomes particularly relevant, and how communication can be optimized. This reflects the first theme, *acknowledgment*, which emphasizes recognizing family members as partners in care and fostering meaningful dialogue to clarify roles and expectations.

Items from the *family as a resource in nursing care* subscale revealed that family members value their presence in the care process. These insights shaped the interview guide to examine why their presence is important, the specific areas they wish to be involved in, and how their involvement may vary based on circumstances. These findings align with the second theme, *alignment*, highlighting family members’ willingness to share information and align their roles with healthcare professionals as partners in care.

Responses to the *family as its own resource* subscale were often neutral, prompting further exploration during the interviews. The interview guide was adapted to encourage participants to reflect on their own needs and how healthcare professionals could support them. This perspective aligns with the third theme, *collaboration*, emphasizing the critical role of family members as collaborators during hospitalization and underscoring the importance of healthcare professionals providing support to ensure their effective involvement in patient care.

## Discussion

Utilizing a sequential explanatory mixed-method design, we gained insights into family members’ opinions regarding their involvement during hospitalization. The acknowledgement of family members as partners in the care process is essential. This acknowledgement not only validates the relevant roles and contributions of family members but also fosters a collaborative environment. While direct patient care remains a priority of care, there is a growing awareness that guidance and attention to the wellbeing of family members by healthcare professionals are also important. This awareness is crucial for maintaining sustainable collaboration in the care process during hospitalization and the preparation for discharge and transfer of care.

In our study, family members expressed a desire to contribute during hospitalization; however, their involvement is still infrequently solicitated by health care professionals. This indicates that the potential of family members as a valuable resource remains unrecognized and underutilized. Given the societal changes and challenges in current health care, this represents a missed opportunity. Healthcare professionals should actively involve family members during hospitalization. As Wittenberg et al. [13] describe, acknowledging the pivotal role of families fosters a strong collaborative relationship between healthcare professionals and family members, which can be facilitated through regular communication [28].

For family members to effectively contribute to patient care and become valuable collaborators, alignment in roles, tasks, and information sharing is essential. Dixe et al. [29] emphasize the importance of key elements such as information sharing, clear process descriptions, and task division based on the knowledge, preferences, and skills of all involved. For example, regular family meetings during admission and throughout the hospitalization process, facilitated by healthcare professionals, can ensure that all parties are aligned on the patient’s care plan, progress, and task division [30, 31].

The emotional responses of several family members during interviews highlight the profound impact the hospital period had on them. This underscores the importance of paying close attention to the wellbeing of family members and coordinating care tasks for the patient in a manner that is supportive to family members. As described by Utz and Warner [7], ensuring the wellbeing of family members requires a supportive environment, which in turn is crucial for effective collaboration and enhancing the overall quality of care provided.

Family members in this study indicated that their situations were unique due to the patients’ interactions with numerous healthcare providers because of multimorbidity, which made care particularly complex. In these situations, family members felt an increasing responsibility for maintaining an overview and coordinating care on behalf of the patient. They expressed a desire to share this responsibility, including managing information processes and maintaining a comprehensive view of the patient’s medical, psychological, and social wellbeing. Given the changing demographics, where older and vulnerable patients with multimorbidity becoming increasingly common, a stronger emphasis should be placed on care coordination within the healthcare system [32]. For example, establishing a central point of contact for both the patient and their family can streamline communication between various healthcare professionals. This ensures that all parties are well informed, and that the patient’s care plan and the division of roles are consistently followed. Implementing such coordination strategies can better support families and enhance the quality of care provided to patients, especially those with multimorbidity.

### Strengths and limitations

In this study, we chose a sequential explanatory mixed-methods design, where quantitative data collection was followed by qualitative data collection. This approach provided deeper insights into how family members wish to be involved in care and their perspective on support for themselves, aspects that were not fully captured through the questionnaire alone.

Although the 52% response rate is typical for studies of this nature and the smaller sample size may limit generalizability, the diversity in responses across wards, combined with the mixed-methods approach, enhances the robustness of the findings.

We distributed the FINC-FO questionnaire widely across the hospital to obtain a broad understanding of the involvement of various family members in patient care. However, questionnaires generally offer limited insights. Further exploration through interviews revealed that families use different terminology to describe involvement compared to the subscales used in the FINC-FO questionnaire. These discrepancies can be attributed to the fact that the FINC-FO questionnaire was originally developed from a nursing perspective [5], resulting in subscales that may not fully align with the language and conceptualization of involvement used by family members.

The adapted FINC-FO questionnaire demonstrated excellent internal consistency but also appears to have some limitations. While family members expressed a desire to be invited to participate at different stages of the care process, spend dedicated time with healthcare professionals, and be involved in decision-making, the FINC-FO does not report how family members envision their roles during these interactions. Additionally, it does not identify the importance of remaining informed throughout the hospitalization, beyond family members providing information to the health care team. The questionnaire also failed to clearly capture family members’ willingness to actively participate in caregiving, provided that their participation is appropriately aligned with the health care team. Scores on the subscale *family as its own resource* were moderate, indicating that this aspect is not perceived as highly relevant. However, further exploration with interviews revealed that family members value being asked “How are you doing?” which indicates an increased awareness of their role in the care process.

Participation in both the questionnaire and interview was voluntary, which may have introduced bias in the findings. Voluntary participation favors a higher response rate from family members who are inclined to caregiving and have particular interest in the topic at hand, while those who are overburdened may have been underrepresented.

The absence of a specific time frame for approaching family members during hospitalization, combined with the unrecorded time interval between discharge and interviews, may have influenced the depth of insights of family members’ perspectives, as their reflections and recall on involvement could change over time.

Because the questionnaire was distributed only in the Dutch language and nearly all respondents indicated the Netherlands as their country of origin, conclusions are not generalizable to families from different ethnic or racial backgrounds.

### Recommendations for further research

Future research should explore the dynamics of family involvement by comparing patients' and family members' perspectives, recognizing the patient as the primary decision-maker. Additionally, studies are needed to develop and test interventions aimed at improving family involvement during hospitalization and facilitating acknowledgement, alignment, and collaboration between patients, family members, and healthcare professionals. Research should examine the changing roles of both family members and healthcare professionals in such interventions, including how family members can take on more active roles and how healthcare professionals can support these roles.

Comparative studies across different cultural contexts should examine factors such as familial hierarchy, communication norms, and healthcare system structures to provide valuable insights into how family roles in healthcare may vary depending on ethnic and/or racial background and to determine which practices can by universally applied.

The FINC-FO requires further development and psychometric testing to establish a robust tool for enriching protocols among healthcare professionals, patients, and family members regarding the coordination of care. The questionnaire could be adapted to incorporate the terminology used by family members as identified in our model and supplemented with specific questions regarding family members’ perceptions of their roles across different stages of hospitalization and their specific information needs. Additionally, questions should explore family members’ willingness and the way they actually want to participate in care.

### Implications for policy and practice

Family members have expressed the need to be acknowledged as partners in care during hospitalization. As such, practical strategies and hospital policies should be developed and implemented to formally acknowledge and integrate family members as essential partners within the healthcare team. The model developed in our research can serve as a tool for raising awareness and as a foundational framework for establishing concrete learning objectives that foster a deeper understanding and practice of patient- and family-centered care.

This approach includes developing training programs for healthcare professionals to enhance their skills in family-centered care and providing resources and tools to help family members clarify their roles in the hospital setting. Hospitals should be encouraged to establish protocols for regular family meetings and collaborative care planning sessions to ensure the continuous and effective involvement of family members. Furthermore, it is crucial to implement ongoing evaluations of the collaborative care efforts, involving all stakeholders, to maintain and enhance the quality of patient care.

## Conclusions

Family members of hospitalized patients wish to be involved in care and acknowledged by healthcare professionals as partners in care. Role definition and information sharing during hospital care should be aligned to achieve effective collaboration between family members and healthcare professionals.

The model developed in this study can serve as a foundation for raising awareness and guiding further research to optimize family involvement during hospitalization.

## Supplementary Information

Supplemental Material 1. FINC-FO questionnaire hospital.

Supplemental Material 2. Interview guide.

## Data Availability

The datasets generated and analysed during the current study are available in the Dataverse repository [18], https://doi.org/10.34894/X4Z40V.

## Abbreviation

FINC-FO: Family Importance in Nursing Care–Families’ Opinions
