# Supplementary material for: Families’ opinions about their involvement in care during hospitalization: a mixed-methods study
Source: BMC Nurs. 2025 Jan 8;24:25. doi: 10.1186/s12912-024-02664-8 (PMC11707841; doi:10.1186/s12912-024-02664-8)
Supplement: Supplementary file 1 — Supplemental Material 1. FINC-FO questionnaire hospital. [file 12912_2024_2664_MOESM1_ESM.pdf]

Family Importance in Nursing Care – Families Opinions (FINC-FO) Questionnaire.

Your Role in the Care of Your Family Member and Collaboration with Healthcare Professionals

The questionnaire consists of a number of general statements about your role as a family member in the care process. Some statements may seem similar but are not identical.
Please select the box with the number that best reflects **your view** of each statement.

Response Categories
1 = Strongly disagree
2 = Disagree
3 = Neutral
4 = Agree
5 = Strongly agree

The Subscales:

● 9 statements about Family as a Resource in Care (1 trough 9)
● 7 statements about Family as a Conversational Partner (10 trough 16)
● 4 statements about Family as its Own Resource (17 through 20)

	**Statements**	**Strongly disagree**	**Disagree**	**Neutral**	**Agree**	**Strongly Agree**
1	My presence when my family member receives care is meaningful.	1	2	3	4	5
2	My presence as a family member eases the workload of nurses and physicians.	1	2	3	4	5
3	It is important to me that I am invited to take an active part in the planning of care for the client (who does what and when)	1	2	3	4	5
4	It is important to me that I am present when care is provided	1	2	3	4	5

5	It is important to me to discuss how I can take an active part in care	1	2	3	4	5
6	It is important to me to be involved in the decision-making process regarding diagnosis and treatment	1	2	3	4	5
7	It gives me a feeling of being useful when I am involved in care.	1	2	3	4	5
8	I possess a lot of worthwhile knowledge about my family member that nurses and physicians can use in their work.	1	2	3	4	5
9	It is important to me that nurses and physicians spend time with me	1	2	3	4	5
10	It is important to me that nurses and physicians know who the patient's family members are.	1	2	3	4	5
11	It is important to me that I am invited to take an active part in caring for my family member.	1	2	3	4	5
12	It is important to me that I am invited to a conversation at the start of care.	1	2	3	4	5
13	A conversation with me as a family member at the start of care will save nurses and physicians time in their work in the future.	1	2	3	4	5
14	It is important to me that I am invited to a conversation at the end of care.	1	2	3	4	5
15	It is important to me that I am invited to a conversation when my family member's situation changes or takes a turn for the worse.	1	2	3	4	5
16	It is important to me that I am regularly invited to a conversation on the progress (planning) of care.	1	2	3	4	5

17	It is important to me that nurses and physicians ask me how they can support me.	1	2	3	4	5
18	It is important to me that nurses and physicians encourage me to cope with the situation myself as best as I can (e.g. by reading books, looking things up on the internet, talking to someone I know, etc.).	1	2	3	4	5
19	It is important to me that nurses and physicians see me as a cooperating partner.	1	2	3	4	5
20	It is important to me that nurses and physicians help me cope with the situation as best as I can.	1	2	3	4	5

Any comments/additions ……………………………………………………………..
..…………………………………………………………………………………………
..…………………………………………………………………………………………
………………………………………………………………………………………
…………………………………………………………………………………….…
………………………………………………………………………………………
………………………………………………………………………………………
………………………………………………………………………………………
…………………………………………………………………………………….…

For this study, we would like to conduct additional interviews on the topic of family involvement in the hospital. Would you be willing to participate in an interview? Interviews will be scheduled at a time and place convenient for you.

☐ Yes, the researcher may contact me for an interview (please provide your email address or phone number below).
☐ No, please do not contact me for an interview.

Email address:<br><br>Phone number:

Would you like to be informed about the results of the study?

☐ Yes, my email address is ………………………………………………
☐ No
