# Supplementary material for: Families’ opinions about their involvement in care during hospitalization: a mixed-methods study
Source: BMC Nurs. 2025 Jan 8;24:25. doi: 10.1186/s12912-024-02664-8 (PMC11707841; doi:10.1186/s12912-024-02664-8)
Supplement: Supplementary file 2 — Supplemental Material 2. Interview guide. [file 12912_2024_2664_MOESM2_ESM.pdf]

Interview guide – Family members' opinion about their involvement during hospitalization

**Subscale FINC-FO**	**FINC-FO questionnaire items**	**Possible interview questions**
family as a resource in nursing care	• My presence is meaningful.<br>• My presence eases the workload.<br>• It gives me a feeling of being useful when I am involved in care.<br>• It is important to me that nurses and physicians spend time with me.	What does involvement mean to you?<br>And during hospitalization?<br>What is important?<br><br>What factors play a role in this (relationship, position within the patient, patient vulnerability, norms and values,...)?
family as a resource in nursing care	• It is important to me that I am invited to take an active part in the planning of care.<br>• It is important to me to be involved in the decision-making process regarding diagnosis and treatment.<br>• I possess a lot of worthwhile knowledge about my family member	*Which role(s) do you envision for the family during hospitalization regarding involvement?*<br><br>***Information resource***<br><br>What information do you want to receive?<br><br>What information do you wat to provide?<br><br>At which moments?<br><br>Why is this important and for whom?<br><br><br>*Role of patient, physicians and nurses in information sharing?*
family as a conversational partner	• It is important to know who the patient's family members are.<br>• It is important to me that I am invited to a conversation at the start	***Conversational partner***<br><br>When are conversations important and with whom?<br>What should be discussed?

	• A conversation with me at the start of care will save time<br>• It is important to me that I am invited to a conversation at the end of care.<br>• It is important to me that I am invited to a conversation when situation changes.<br>• It is important to me that I am regularly invited to the progress (planning) of care.	What is the goal/importance?<br><br>*Role of patient, physicians and nurses in conversations?*
family as a resource in nursing care<br><br>*family as a resource in nursing care*<br><br>*family as a conversational partner*	• It is important to me that I am present when care is provided.<br><br>• It is important to me to discuss how I can take an active part in care.<br><br>• It is important to me that I am invited to take an active part in caring for my family member.	**Providing care** during hospitalization.<br><br>How do you perceive this?<br><br>In what form and at what moments?<br><br>What is important in this regard?<br><br>How to align (wishes and expectations)?<br><br>*Role of patient, physicians and nurses providing care by family members?*
family as its own resource	• It is important to me to ask how they can support me.<br>• It is important to me that they<br>• encourage me to cope with the situation myself as best as I can.<br>• It is important to me to help me cope with the situation as best as I can.<br>• It is important to me that healthcare professionals see me as a cooperating partner.	Do you find it important that healthcare professionals also take your needs as a family member into account?<br><br>At certain moments/situations?<br><br>In what way? (e.g., asking how are you doing)
